# Acute, isolated fractures of the metatarsal bones: an epidemiologic study

**DOI:** 10.1007/s00402-022-04396-3

**Published:** 2022-03-02

**Authors:** Viktoria Herterich, Luzie Hofmann, Wolfgang Böcker, Hans Polzer, Sebastian Felix Baumbach

**Affiliations:** Department of Orthopaedics and Trauma Surgery, Musculoskeletal University Center Munich (MUM), University Hospital, LMU Munich, Ziemssenstr. 5, 80336 Munich, Germany

**Keywords:** Metatarsal, Fracture, Epidemiology, Fracture distribution

## Abstract

**Introduction:**

Although metatarsal fractures are common, the significance of previous epidemiologic studies is limited to specific fracture entities, subpopulations, or heterogeneous fracture aetiologies. The aim of the study was to assess the epidemiology of isolated metatarsal fractures in an adult population at a level-1 trauma centre.

**Materials and methods:**

Radiological and clinical databases were searched for a five-year period. Eligible were all patients with acute isolated metatarsal fractures over the age of 18 years with radiographs in two planes available. Stress fractures, injuries affecting Lisfranc joint stability, and concomitant injuries to other regions than the metatarsals were excluded. Data collection included general demographics, mechanism of injury, season of the trauma and fracture details.

**Results:**

Out of 3259 patients, 642 patients met the inclusion criteria and were included for the analysis. The patients’ mean age was 44.5 ± 18.9 years, 50.6% were female. 83.3% suffered an isolated, 16.7% multiple metatarsal fractures. Single metatarsal fractures occurred predominantly at the fifth metatarsal bone (81.3%), their frequency decreased with increasing age, with a seasonal peak during the summer. Patients suffering multiple metatarsal fractures were significantly older (51.6 ± 21.2 vs. 43.0 ± 18.1 years; *p* < 0.001) and the injury resulted significantly more often from a high-energy trauma (6.7% vs. 23.4%; *p* < 0.001). Multiple metatarsal fractures occurred evenly throughout all metatarsals but revealed a focus on female population with no seasonal differences.

**Conclusion:**

Single metatarsal fractures predominantly occurred at the fifth metatarsal bone and showed a seasonal, gender and age dependency. Multiple metatarsal fractures were homogeneously distributed between the different metatarsals with distinct age-dependent gender differences.

**Level of evidence:**

Level III.

## Introduction

Metatarsal fractures belong to the ten most common fractures with a prevalence of 3.2–6.8% of all fractures with an annual incidence of 67–75.4/100.000 per year [3, 7, 17, 21]. Furthermore, they account for up to 88.5% of all fractures to the foot [8, 22, 23]. Despite their frequency, epidemiological data on metatarsal fractures are rare, as most recent literature focusses on treatment recommendations, especially for the base of the fifth metatarsal and lacks epidemiological data [1, 9, 11, 15].

The significance of previous epidemiological studies on metatarsal fractures is limited as they either focused on specific entities (fractures to the base of the fifth metatarsal or dancer’s fractures), evaluated subpopulations only (elderly Caucasian women or children), or included heterogeneous fracture aetiologies (including concomitant injuries/stress fractures) [2, 4, 5, 8, 13, 16, 17, 19, 21]. Consequently, we are still missing valid epidemiological data of a well-characterized cohort for one of the most common fractures in adulthood.

Therefore, the aim of this study was to assess the epidemiology of isolated metatarsal fractures in an adult population over a 5-year period at a level-1 trauma centre.

## Materials and methods

The herein presented work is a retrospectively radiographic-epidemiological study. The study was approved by the local ethics committee (#20.0442).

### Patient screening

The hospital’s clinical database was searched per the ICD-10 S92.0 code and the radiological database per the search terms: (“metatars*” OR “midfoot*”) AND (“fracture” OR “bon* injury”) between 01.01.2015 and 12.31.2019. Inclusion criteria were adult patients (≥ 18 years) with acute isolated metatarsal fracture(s) and plain radiographs of the foot in two planes available within the first 14 days of the injury. Exclusion criteria were stress fractures, defined as slight fractures without relevant trauma, injuries affecting Lisfranc joint stability, and any concomitant injury to other regions than the metatarsals.

### Patient selection

The results of the clinical and radiological database were merged, and duplicates were removed. The removal of the duplicates was performed after merging the results; therefore, it remains unclear if the duplicates were within each database or between both databases. The resulting database was screened for eligible patients, independently by two blinded investigators (VH, LH). In case of disagreement, the conflict was resolved by discussion with the senior author (SFB).

### Data collection

From all eligible patients, the following data points were collected: general demographics, mechanism of injury (high energy/low energy)—high energy was defined as every trauma mechanism beyond supination force on plane ground—season of accident, and fracture details including side fracture, number of metatarsals fractured, and fracture location (proximal, shaft, distal).

### Statistics

Next to general demographics, group comparisons were conducted using Pearson Chi-Square, independent sample t-test, oneway ANOVA with Tukey post hoc analysis were appropriate. Due to multiple testing, a Bonferroni correction was conducted setting the level of significance to *p* < 0.01. Data are presented as mean ± standard deviation, if not stated differently. All statistics were computed using SPSS (26.0, IBM).

## Results

**Fig. 1:**
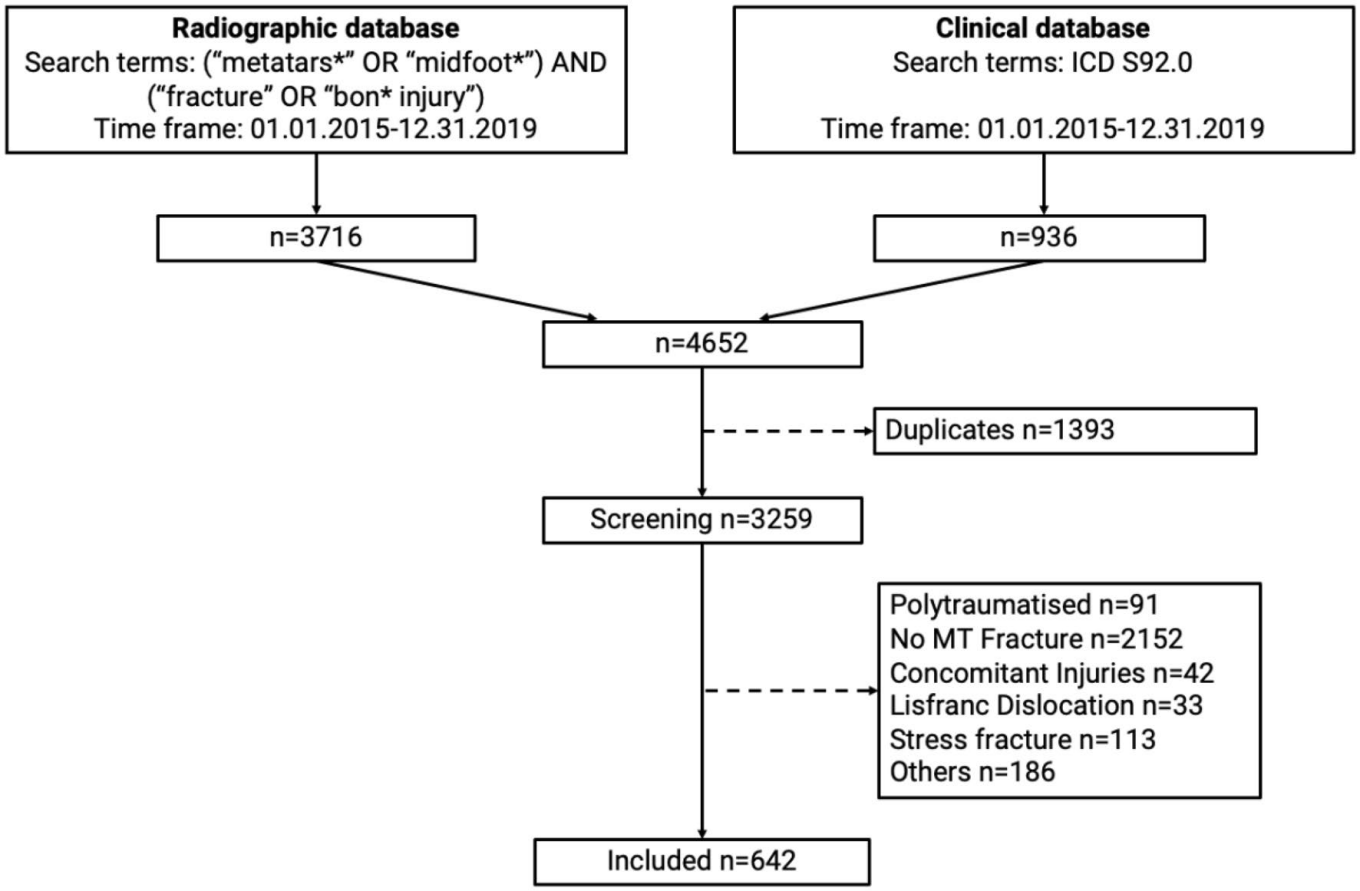
Patient selection flow chart. *n* number, *MT* metatarsal. Other reasons: delayed presentation; missing initial X-ray; date of Injury; > 2019/< 2015

In the radiographic database, 3716 patients were identified and in the clinical database, 936 patients were identified. Removal of duplicates (*n* = 1393) resulted in 3259 patients. Following independent, blinded review, 642 patients were eligible for final analysis according to the inclusion criteria (Fig. 1).

The patients’ mean age was 44.5 ± 18.9 years, 50.6% were female, and the left side was fractured in 50.2% of patients. An overview of the general demographics and fracture distribution is provided in Table 1. 535 patients (83.3%) suffered an isolated metatarsal fracture. In 16.7% of the patients, more than one metatarsal bone was fractured. Patients with multiple metatarsal fractures were significantly older (51.6 ± 21.2 vs. 43.0 ± 18.1 years; *p* < 0.001) and the injury resulted significantly more often from a high-energy accident (6.7% vs. 23.4%; *p* < 0.001) compared to patients with a single metatarsal fracture. No significant differences were found for sex (*p* = 0.459) or the fractured side (*p* = 1).

**Table 1:** General demographic overview of different fracture combination and distributions

Number of metatarsals fractured	Number of patients [percentage^a^]	Most common combination [percentage^b^]	Age [mean ± SD]	Sex [% female]	Side [% left]	Percent high energy
1	535	–	43.0 ± 18.1	49.9%	50.1%	6.7
2	61 [57.0%]	IV + V [45.9%]	54.7 ± 21.7	62.3%	45.9%	14.8
3	32 [29.9%]	II + III + IV [58.3%]	42.6 ± 18.8	43.8%	46.9%	28.1
4	13 [12.1%]	II + III + IV + V [68.8%]	57.2 ± 22.7	38.5%	76.9%	46.2
5	1 [0.9%]	–	[77]	[100%]	[100%]	[100%]

^a^Percentage of total multiple fractured MT (*n* = 107)

^b^Percentage within that group

**Fig. 2:**
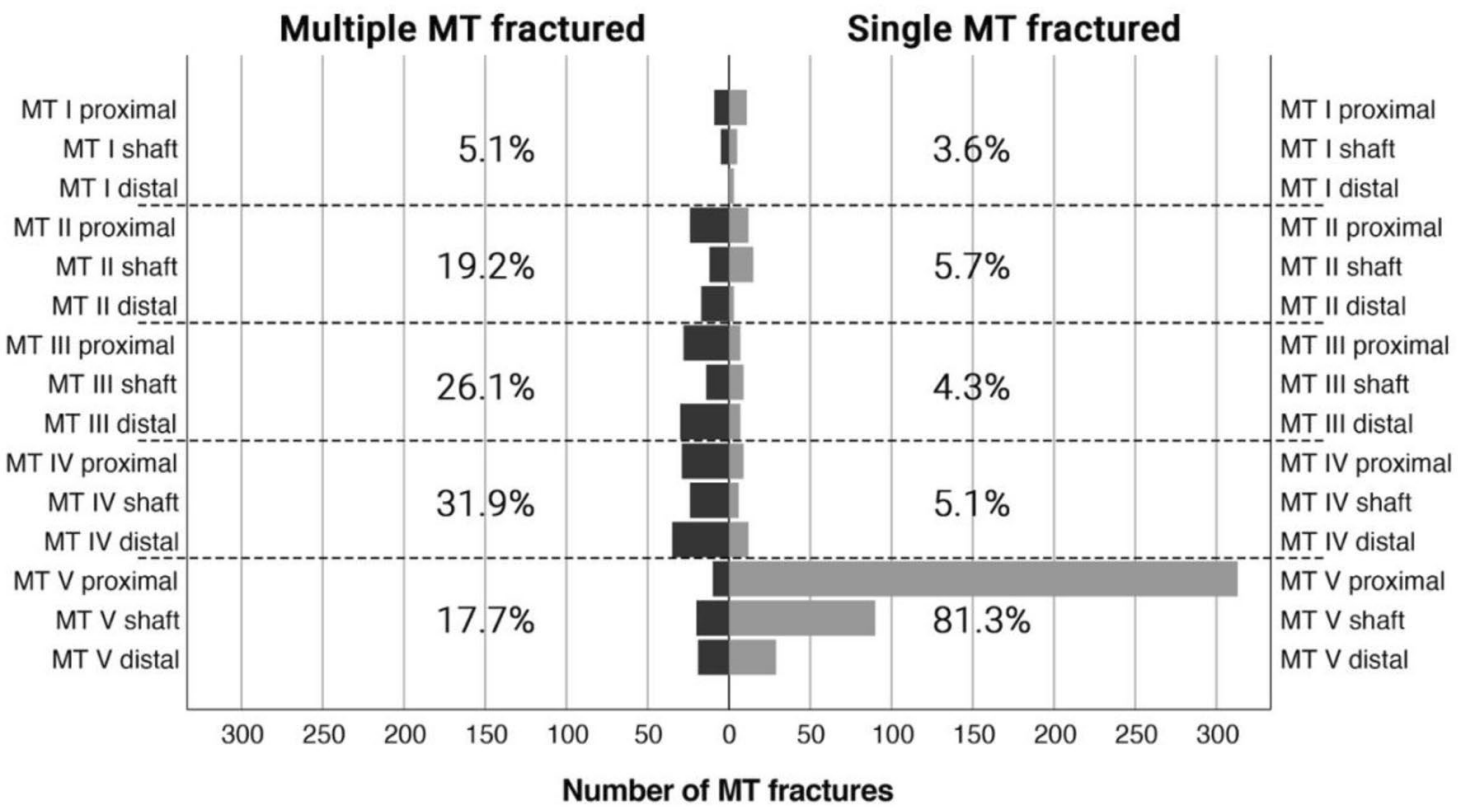
Distribution per the number of metatarsals fractures. *MT* metatarsal

Out of all fractures, 4.1% occurred at the first metatarsal (MT), 10.3% at MT II, 11.8% at MT III, 14.3% at MT IV, and 59.6% at MT V. The fracture distribution (proximal, shaft, distal) per the number of the metatarsals fractured (single/multiple) differed significantly (*p* < 0.001) and is outline in Fig. 2. Whereas multiple metatarsal fractures showed a rather homogeneous distribution, single metatarsal fractures occurred predominantly (81.3%) within the fifth metatarsal bone.

Out of all isolated fifth metatarsal fractures, 72.5% occurred proximal, 20.8% in the shaft region and 6.7% distally. Fracture locations within the fifth metatarsal were independent of age (*p* = 0.083), sex (*p* = 0.171), fractured side (*p* = 0.355), or trauma mechanism (*p* = 0.012). Regarding the proximal fractures to the fifth metatarsal, the distribution according to the Lawrence and Botte classification was: Type I: 63.0%, Type II: 21.8%, Type III: 15.2% [12].

**Fig. 3:**
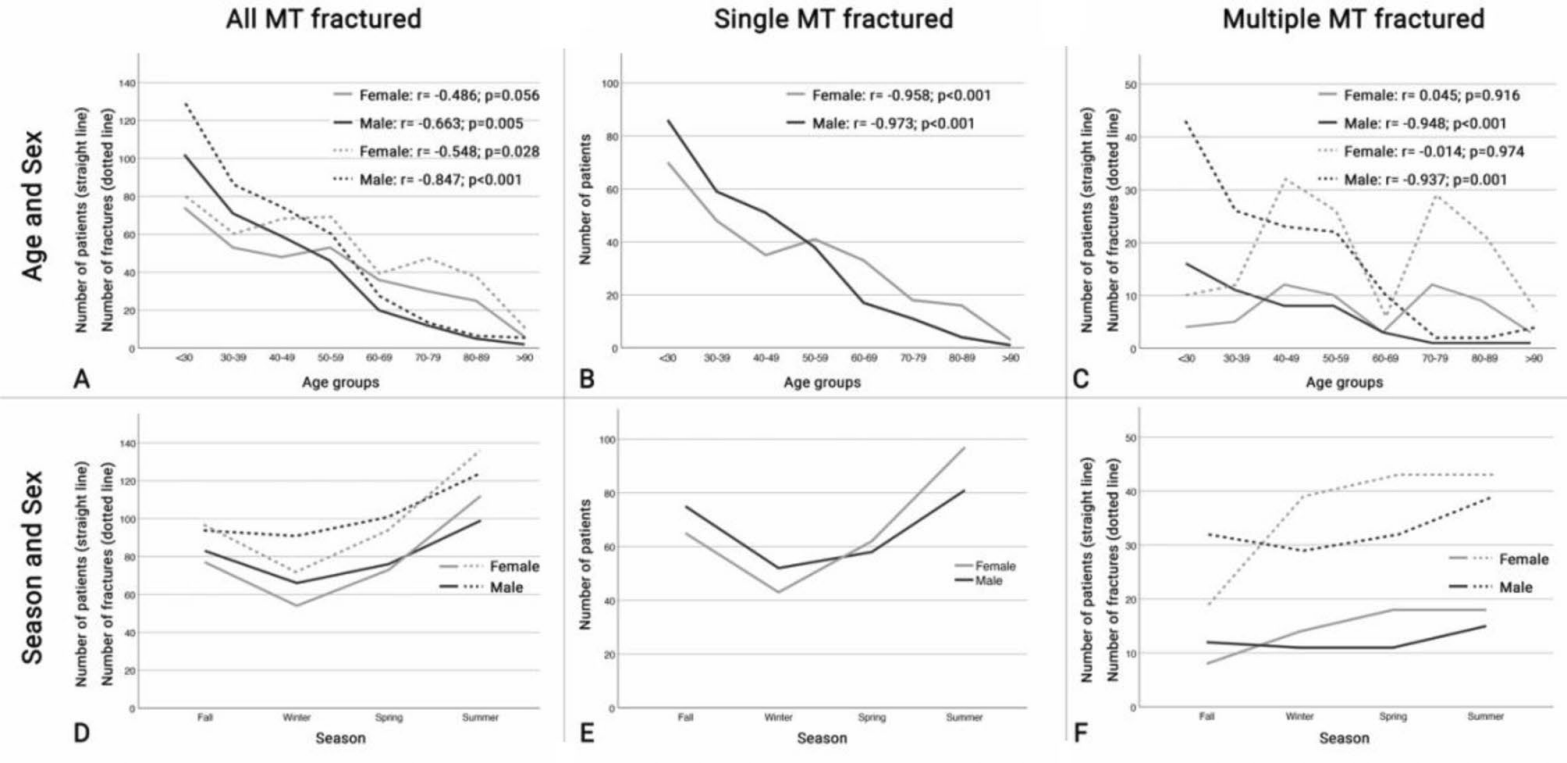
Gender-specific age and season distribution of metatarsal fractures. **A** Age- and sex-specific distribution of single metatarsal fractures; **B** Age- and sex-specific distribution of multiple metatarsal fractures; **C** Season- and sex-specific distribution of single metatarsal fractures; **D** Season- and sex-specific distribution of multiple metatarsal fractures

Next, the age distribution was analysed. Overall, a moderate negative correlation was found between age and the number of patients (*r* = − 0.581; *p* < 0.001) or the number of metatarsal fractures (*r* = − 0.712; *p* < 0.001). This moderate negative correlation stayed true when conducting the gender specific analysis (Fig. 3A). Interestingly, there was a higher degree of correlation for male compared to female patients, both when analysing the number of patients and the number of metatarsal fractures.

Then, the dataset was analysed separately for single or multiple metatarsal fractures and their correlation to age. For single metatarsal fractures, an overall high correlation was found (*r* = − 0.951; *p* < 0.001), which stayed true when analysed separately for gender (Fig. 3B). For multiple metatarsal fractures, a moderate negative correlation was found for age and both, the number of patients (*r* = − 0.526; *p* = 0.036) as well as the number of fractured metatarsals (*r* = − 0.544; *p* = 0.029). Interestingly, again a high negative correlation was found for male patients and the number of patients and the number of fractures (Fig. 3C). No correlation was observed for female patients and for the number of patients or fractures. Observationally, female patients rather showed two age peaks, one between 40 and 49 years and the other between 70 and 79 years.

Finally, the season distribution for metatarsal fractures was investigated. Looking at all metatarsal fractures (Fig. 3D), there appears to be a gender-independent peak during summer and a low during winter. A similar trend was found for single metatarsal fractures (Fig. 3E). When looking at multiple metatarsal fractures, a more homogeneous distribution was observed with trending lower numbers during fall and winter when compared to spring and summer (Fig. 3F).

## Discussion

This study is the first to investigate the epidemiology of isolated metatarsal fractures in a large adult population. Significant differences were found for the anatomical location, age, and seasonal distribution between single and multiple fractured metatarsals. Single metatarsal fractures showed a distinct age-dependent decrease and a peak during summer. Multiple metatarsal fractures revealed a sex dependent age distribution, with fractures in females occurring predominantly between 40–49 and 70–79 years.

The presented dataset of 624 adult patients suffering an isolated metatarsal fracture compares favorably to literature. Previous epidemiologic studies comprised around 400 patients and furthermore presented heterogeneous patient populations [2, 17]. An exception could be the study by Zhao et al. [24] who apparently included 1949 adult metatarsal fractures. Unfortunately, the study was published in Chinese and could therefore not be reviewed.

The overall distribution of metatarsal fractures reported herein, i.e., a rather homogenous distribution between MT I and IV fractures and a clear peak in MT V fractures has been stated before [2, 17, 18]. When analyzing the whole sample, 59.6% of metatarsal fractures were located at the fifth metatarsal, which is well in line with data published previously, varying between 43 and 68% [6, 10, 17, 18, 20, 24]. Interestingly, when subdividing the whole population between multiple and single metatarsal fractures, only 17.7% of all multiple but 81.3% of single metatarsal fractures occurred at the fifth metatarsal.

Furthermore, also the fracture location distribution (proximal—shaft-distal) within the fifth metatarsal bone varied between single- and multiple-metatarsal fractures. Previous studies have reported a dominance of proximal metatarsal fractures, ranging from 49.8 to 72% of all MT V fractures [6, 10]. When conducting this analysis separately for single and multiple metatarsal fractures, we found a rather homogeneous distribution for multiple MT fractured but a predominance of proximal MT V fractures for isolated MT fractures.

The authors are aware of two studies only, which discriminated single and multiple metatarsal fractures [2, 17]. They reported that 7.8–15.6% of all metatarsal fractures affected multiple metatarsals. However, these studies did not perform further analyses. Due to the herein observed differences between single and multiple metatarsal fractures, future epidemiological studies should incorporate not only a plane description of fracture frequency but also possible differences between different fracture etiologies and patterns.

Next, the influence of the patients’ age on the occurrence of metatarsal fractures was analyzed. Previous studies have reported an age-dependent decrease in metatarsal fractures [2, 3, 10, 17, 20]. Moreover, they have stated an additional age- and gender-dependent distribution. For male patients, metatarsal fractures were reported to peak in their thirties. For female patients’ metatarsal fractures were found to peak between the age 50 and 70 years [2, 10, 14, 18]. One major limitation of these studies was again, that they did not discriminate between single and multiple metatarsal fractures. In the herein published cohort, a comparable trend was observed when analyzing the whole sample (Fig. 3A). Fractures in male patients showed a constant decrease in patients aged 30 years and above. Fractures in female patients showed two peaks, at the ages of 50–59 years and 70–79 years. When differentiating single and multiple metatarsal fractures, one does see a different, but more congruent picture. In single metatarsal fractures, a high negative correlation was found between age and both, male (*r* = − 0.973; *p* < 0.001) and female (*r* = − 0.958; *p* < 0.001) patients (Fig. 3B). For multiple metatarsal fractures, again a high negative correlation was found between age and number of patients (*r* = − 0.948; *p* < 0.001)/of metatarsal fractures (*r* = − 0.937; *p* = 0.001) in male patients. But no correlation between age and female number of patients (*r* = 0.045; *p* = 0.916)/metatarsal fractures (*r* = − 0.014; *p* = 0.974), but rather two clear peaks between 40–49 years and 70–79 years. This age and gender-dependent distribution has considerable implications for the clinical routine as well as future studies. Whereas single metatarsal fractures apparently show an age-dependent decrease, multiple metatarsal fractures in female show peaks similar to those in osteoporosis-associated fractures. Therefore, one can hypothesis, that female patients suffering multiple metatarsal fractures might be at higher risk for osteoporosis.

When investigating the season-dependent fracture distribution, the differentiation between single and multiple metatarsal fractures again proofed important. Whereas the cumulative (Fig. 3D) and single metatarsal fracture (Fig. 3) analysis showed a peak for the summer and low for the winter, a considerably more homogeneous distribution was found for multiple metatarsal fractures. This again raises the suspicion, that pathomechanisms of isolated multiple metatarsal fractures might be different to those of single metatarsal fractures. Explanation could be the above mentioned, age dependent, alteration in bone metabolism, i.e., osteoporosis or the higher number of high energy accidents. Further studies are needed to proof the hypothesis that metatarsal fractures could be osteoporosis-associated fractures.

### Limitations of the study

The major limitation of this study is its descriptive design. Still, for the first time, we were able to provide a comprehensive picture of the epidemiology of isolated metatarsal fractures in an adult population.

Furthermore, the study has no direct clinical implementation. But researchers face a similar problem when conducting descriptive or retrospective studies. Still, the detailed epidemiological analysis of metatarsal fractures does not only have an academic purpose but does have considerable implications for future studies. As outlined above, future studies should investigate a possible correlation between multiple isolated metatarsal fractures and osteoporosis. Considering their early age peak between 40 and 49 years, they might be identified as early indicator fractures for osteoporosis.

### Strengths of the study

First to mention is this study’s clearly defined patient population assessing only isolated metatarsal fractures in adult patients and excluding all concomitant ligamentous and bony injuries. Therefore, a more decisive statement can be made about the thus very determined fracture types. Furthermore, the review of patients was performed by two independent reviewers and conflicts were solved by consulting the senior author; therefore, a very strict and even patient selected was achieved.

Finally, a detailed analysis of the fracture patterns was performed. Looking at the results reported, it seems extremely important to differentiate between single and multiple MT fractures as they differed significantly.

## Conclusion

This is the largest epidemiological studies on isolated metatarsal fractures in adult patients. Based on the data available, a differentiation between the two entities single and multiple isolated metatarsal fractures seems necessary. Whereas single metatarsal fractures predominantly occur at the fifth metatarsal bone and show a seasonal dependency, multiple metatarsal fractures revealed a rather homogeneous distribution between the different metatarsal bones with distinct age- and gender-dependent differences. Future studies should not only emphasis the differentiation between single and multiple isolated metatarsal fractures, but also investigate a possible correlation between multiple isolated metatarsal fractures in female patients and osteoporosis.
