# Correction to: Acute, isolated fractures of the metatarsal bones: an epidemiologic study

**DOI:** 10.1007/s00402-023-04784-3

**Published:** 2023-01-31

**Authors:** Viktoria Herterich, Luzie Hofmann, Wolfgang Böcker, Hans Polzer, Sebastian Felix Baumbach

**Affiliations:** Department of Orthopaedics and Trauma Surgery, Musculoskeletal University Center Munich (MUM), University Hospital, LMU Munich, Ziemssenstr. 5, 80336 Munich, Germany

**Correction to: Archives of Orthopaedic and Trauma Surgery** 10.1007/s00402-022-04396-3

The original version of this article unfortunately contained a mistake. All authors’ first name and last name have been swapped.

Here is the correct first name and last name:

- Viktoria Herterich
- Luzie Hofmann
- Wolfgang Böcker
- Hans Polzer
- Sebastian Felix Baumbach

The original article has been corrected.

